# A eudicot *MIXTA* family ancestor likely functioned in both conical cells and trichomes

**DOI:** 10.3389/fpls.2023.1288961

**Published:** 2023-12-18

**Authors:** Simra Zahid, Anjelique F. Schulfer, Verónica S. Di Stilio

**Affiliations:** Department of Biology, University of Washington, Seattle, WA, United States

**Keywords:** epidermis, MIXTA family, non-core eudicots, polyploidy, trichomes, conical cells, ranunculid, R2R3 MYB

## Abstract

The *MIXTA* family of MYB transcription factors modulate the development of diverse epidermal features in land plants. This study investigates the evolutionary history and function of the *MIXTA* gene family in the early-diverging eudicot model lineage *Thalictrum* (Ranunculaceae), with R2R3 SBG9-A MYB transcription factors representative of the pre-core eudicot duplication and thus hereby referred to as “paleo*MIXTA*” (*PMX*). Cloning and phylogenetic analysis of *Thalictrum paleoMIXTA* (*ThPMX*) orthologs across 23 species reveal a genus-wide duplication coincident with a whole-genome duplication. Expression analysis by qPCR confirmed that the highest expression is found in carpels, while newly revealing high expression in leaves and nuanced differences between paralogs in representative polyploid species. The single-copy ortholog from the diploid species *T. thalictroides* (*TthPMX*, previously *TtMYBML2*), which has petaloid sepals with conical–papillate cells and trichomes on leaves, was functionally characterized by virus-induced gene silencing (VIGS), and its role in leaves was also assessed from heterologous overexpression in tobacco. Another ortholog from a species with conical–papillate cells on stamen filaments, *TclPMX*, was also targeted for silencing. Overexpression assays in tobacco provide further evidence that the *paleoMIXTA* lineage has the potential for leaf trichome function in a core eudicot. Transcriptome analysis by RNA-Seq on leaves of VIGS-treated plants suggests that *TthPMX* modulates leaf trichome development and morphogenesis through microtubule-associated mechanisms and that this may be a conserved pathway for eudicots. These experiments provide evidence for a combined role for *paleoMIXTA* orthologs in (leaf) trichomes and (floral) conical–papillate cells that, together with data from other systems, makes the functional reconstruction of a eudicot ancestor most likely as also having a combined function.

## Introduction

Plants have evolved diverse epidermal features to adapt to their environment (Javelle et al., 2011), and epidermal cell types are useful micromorphological markers of the different plant organs (Cavallini-Speisser et al., 2021). *MYB* genes are a prominent land plant gene family that encode transcription factors characterized by having one to four contiguous repeats, with most plants having two repeats, R2 and R3, that function in secondary metabolite production and cell identity (Stracke et al., 2001). The R2R3 MYB subgroup 9A *MIXTA/MIXTA-like* gene family has specialized roles in epidermal cells (Brockington et al., 2013), including the first characterized function in conical cells (Noda et al., 1994; Baumann et al., 2007; Di Stilio et al., 2009), trichomes (Jakoby et al., 2008; Yan et al., 2018; Qin et al., 2021), and ovule epidermal cells that make cotton fibers (MaChado et al., 2009). Certain *MIXTA-like* orthologs have evolved multiple functions in epidermal cell patterning, such as *Antirrhinum majus AmMYBML1* in regulating trichome branching and conical cells (Pérez-Rodríguez et al., 2005). In addition to the previously reviewed *MIXTA* family functions (Brockington et al., 2013), recent studies further support roles in glandular trichomes of mint (Qi et al., 2022) and *Nepetes* (Lamiaceae, Zhou et al., 2022) as well as in trichome patterning and morphogenesis in tomato leaves (Galdon-Armero et al., 2020). A novel function for *MIXTA* family orthologs in cuticle (Lashbrooke et al., 2015) is further supported in the early land plant model *Marchantia* (Xu et al., 2020) and in the orchid *Phalaenopsis* (a monocot, Lu et al., 2022). A *MIXTA* family ortholog from another orchid, *Dendrobium crumenatum*, is capable of rescuing the branched trichome phenotype of *Arabidopsis thaliana noeck* mutants, even though it does not produce trichomes in the orchid (Gilding and Marks, 2010). Since most of the functional information on *MIXTA* family genes comes from the core eudicots, additional functional studies of *MIXTA* family genes in early-diverging eudicots are warranted to inform the reconstruction of the ancestral gene function of this important transcription factor family in the eudicots, the most species-rich clade containing three quarters of all angiosperm species (Simpson, 2019).

Trichomes or epidermal hairs represent an emerging model for the study of single-cell differentiation that is structurally simple and easily accessible (Yang and Ye, 2013). Trichomes can be non-glandular or glandular (Feng et al., 2021): the former typically provides protection against UV-B radiation and dehydration, while the latter is a warehouse of defense metabolites against herbivores (Jaime et al., 2013; Yan et al., 2018; Chalvin et al., 2021). On the one hand, differentiation of unicellular trichomes is well understood in *A. thaliana*, where it is controlled by an activation–inhibition loop (Jakoby et al., 2008). Positive regulators GLABRA3 (GL3), GLABRA1 (GL1), and TRANSPARENT TESTA GLABRA1 (WD40) form a trimeric complex with basic helix–loop–helix proteins (bHLH) known as MYB-bHLH-WD40 that stimulates trichome differentiation in the epidermis (Pesch and Hülskamp, 2009). In the surrounding cells, transcription factors such as TRIPTYCHON (TRY) and TRICHOMELESS1,2 (TCL1 and TCL2) bind and inactivate the bHLH-WD40 complex, thereby suppressing trichome development (Gao et al., 2017). The regulatory network for multicellular trichomes, on the other hand, is less clear, with HD-ZIP IV transcription factors known to interact with *MIXTA* family orthologs during trichome development in maize, cucumber, and the asterid *Artemisia annua* (Vernoud et al., 2009; Wang et al., 2016; Yan et al., 2018), while a B-type cyclin plays this role in tomato (Gao et al., 2017). Hence, further investigation of trichome development and the underlying genetic regulators is warranted.

*Thalictrum* (meadow-rue) are herbaceous perennials in the family Ranunculaceae that lack petals and are pollinated by insects, the wind, or both, with associated floral morphotypes (Martínez-Gómez et al., 2023). *Thalictrum MIXTA* family orthologs are expressed in the epidermis of floral organs that contain conical–papillate cells (petaloid sepals or showy stamen filaments) that aid in attracting insect pollinators and were previously shown to affect conical–papillate cell elongation when overexpressed in tobacco (Di Stilio et al., 2009). However, the function of these non-core eudicot *MIXTA* orthologs remains untested in *Thalictrum*. As predicted then, this gene family represents a lineage that is sister to the core eudicot *MIXTA/MIXTA-like* duplication (Brockington et al., 2013). We will therefore hereby refer to “paleo*MIXTA*” (*PMX*) following the floral MADS box gene nomenclature (paleo*AP3*; Kramer et al., 1998) and *MX* shortcut for *MIXTA* (Brockington et al., 2013) and distinguish this gene lineage from the core eudicot *MIXTA*/*MIXTA-like*. Based on this new information, the published gene *TthMYBML2* (Di Stilio et al., 2009) is hereby renamed to *TthPMX*, and the others newly described here will follow this nomenclature scheme.

We chose to compare the gene functions in *T. thalictroides*, with conical–papillate cells in their petaloid sepals, and *T. clavatum*, with conical–papillate cells in their showy stamen filaments. The two species are closely related within clade I and represent the ancestral traits for the genus of diploid, hermaphrodite, and insect pollinated (Soza et al., 2012; Soza et al., 2013; Wang et al., 2019). Using virus-induced gene silencing (VIGS) as previously described (Di Stilio et al., 2010), we downregulated the paleo*MIXTA* ortholog in both species. We show evidence for a new role for this floral MYB transcription factor in leaf trichomes and further show that it is necessary for conical cell formation that contributes to petaloidy in sepals and stamens for these two species of *Thalictrum* with distinct floral morphologies.

This study aims broadly to provide functional information for members of the paleo*MIXTA* gene lineage via functional assays in flowers and leaves of early-diverging eudicots to ultimately contribute to an increased understanding of the functional evolution and molecular mechanisms of this important transcription factor family. To that end we (1) reconstruct the evolutionary history of *MIXTA* family orthologs from the genus *Thalictrum*, (2) investigate the function of two paleo*MIXTA* orthologs in the flowers and leaves of two distinct species, and (3) identify candidate genes underlying the *MIXTA* gene regulatory network (GRN) in leaves by RNA-Seq.

The evidence presented here from functional studies of *Thalictrum* paleo*MIXTA* representatives suggests that the function of a putative pre-*MIXTA/MIXTA-like* core eudicot duplication ancestor likely consisted of a combined role in conical–papillate cells and trichomes. This multifunctional eudicot ancestral gene presumably could have parsed out its distinct roles among paralogs after the core eudicot *MIXTA*/*MIXTA-like* duplication as well as within taxa that underwent further lineage-specific duplications. Our finding of a duplication event within *Thalictrum* resulting in differential patterns of paralogous gene expression provides a potential basis for the evolution of increased epidermal trichomes found on the leaves and flowers of derived wind-pollinated polyploid taxa and thus represents an interesting avenue for future research.

## Results

### An early duplication in the *Thalictrum* paleo*MIXTA* lineage coincides with a genus-wide whole-genome duplication

**Figure 1:**
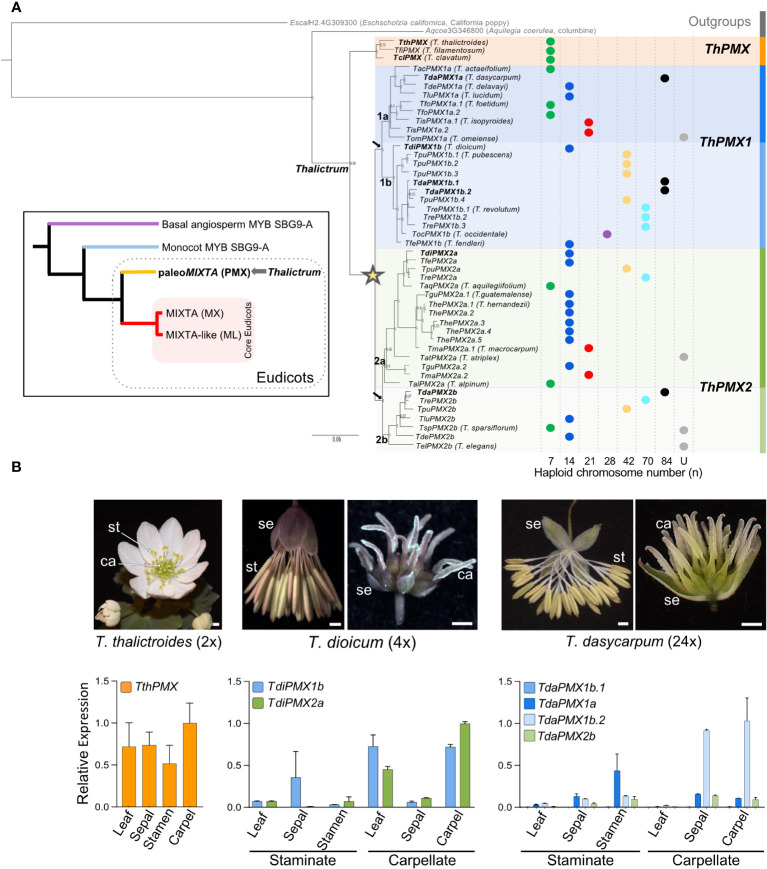
Gene phylogeny of the *Thalictrum MIXTA* family of MYB transcription factors (R2R3 Subgroup 9A). **(A)** Bayesian inference phylogeny with *Aquilegia coeruleae* (Ranunculaceae) and *Eschscholzia californica* as outgroups showing two major clades: clade I (orange) named *Thalictrum* paleo*MIXTA* (*ThPMX*) and clade II with an inferred gene duplication event (star) leading to subclades *ThPMX1* (blue) and *ThPMX2* (green). Another duplication in each clade (black arrows) leads to “a” and “b” subclades, and additional species-level duplications are designated with a period followed by a sequential number (e.g., *TdaPMX1b.1*–*TdaPMX1b.4*). Posterior probabilities are shown next to the nodes. Branch lengths indicate substitutions per site. Haploid (*n*) chromosome numbers are shown for each species as colored dots, from *n* = 7 (diploid) to *n* = 84 (24-ploid); U = unknown (data from Soza et al., 2013). Genes in bold are the subject of this study. Inset: Simplified tree of the *MIXTA* gene family in angiosperms [modified from Brockington et al. (2013)] highlighting the ranunculid *MIXTA* lineage (paleo*MIXTA*) that includes the *Thalictrum* orthologs. Organismal coding: purple = ANITA grade; blue = monocots; yellow = early-diverging eudicots; red = core eudicots. **(B)** Relative expression of *Thalictrum* paleo*MIXTA* in leaves and floral tissues of representative species from major clades. From left to right: Representative photos of hermaphrodite flowers of *T. thalictroides* (diploid) and staminate and carpellate flowers of dioecious *T. dioicum* (tetraploid) and *T. dasycarpum* (high-level polyploid). Quantitative RT-PCR showing the average expression level of *Thalictrum* paleo*MIXTA* orthologs relative to the average expression of two housekeeping genes *ThACTIN* and *ThEEF1-alpha*, normalized to the highest-expressing organ (carpel) and paralog. Color-coding matches the gene lineages shown in **(A)**. Error bars represent ± standard error of the mean. *N* = 4–7 flowers. Photo credits: Samantha Hartogs (*T. thalictroides* and *T. dasycarpum*); Kelsey Galimba (*T. dioicum*). se, sepals; st, stamens; ca, carpels.

A phylogenetic approach was used to investigate the evolutionary history of the *Thalictrum* (Ranunculaceae) MYB SBG9-A *MIXTA* gene family or paleo*MIXTA* (*PMX*, **Figure 1A** inset). To this end, a nucleotide alignment was constructed for 49 MYB SBG9-A *Thalictrum* sequences isolated from 23 species spanning the two main clades (Soza et al., 2012; Martínez-Gómez et al., 2023) and identified based on the conserved R2R3 and Subgroup 9-A domains (**Supplementary Figure S1**). The two SBG9-A outgroups chosen were columbine Aqcoe3G346800 (Ranunculaceae, *Aquilegia coerulea*) and California poppy EscalH2.4G309300 (Papaveraceae, *Eschscholzia californica* var. Aurantiaca Orange King Plant1.1 v1.1, DOE-JGI, http://phytozome.jgi.doe.gov/info/Ecalifornicavar_AurantiacaOrangeKingPlant1_1HAP2_v1_1).

A consensus phylogenetic tree was inferred using the Bayesian optimality criterion implemented in MrBayes (**Figure 1A**). *Thalictrum MIXTA* family sequences form two distinct clades with strong support, coincident with the two major clades previously described: clade I *Thalictrum* paleo*MIXTA* (*ThPMX*) containing only diploid species and clade II with subclades paleo*MIXTA1* (*ThPMX1*) and *ThPMX2* having both diploid and polyploid species. The gene tree suggests a gene duplication event at the base of the two subclades (**Figure 1A**, star), with paralogs from polyploid species distributed among the two resulting clades and instances of potential paralog loss (e.g., the 20-ploid *T. revolutum* has a gene copy in all clades except *ThPMX1a*). Each of these larger gene clades, in turn, underwent a subsequent duplication, designated as “a” and “b”. Certain polyploid species, particularly those with high ploidy level, like *T. dasycarpum* (24x) and *T. revolutum* (20x), had additional species-level single-gene duplications that were consecutively numbered (e.g., *TpuPMX1b.1*-*TpuPMX1b.4*). Thus, *MIXTA* family orthologs only partially follow the species phylogeny, suggesting a reticulated evolutionary history for *Thalictrum* that could not be previously detected when using chloroplast loci and the transcribed spacers ITS and ETS (Soza et al., 2013; Martínez-Gómez et al., 2023).

### Differential expression of *Thalictrum* paleo*MIXTA* paralogs in leaves and floral organs

To broadly investigate the spatial expression pattern of *Thalictrum paleoMIXTA*, we performed qPCR on leaves and dissected floral organs (sepals, stamens, and carpels) of recently open flowers, where expression is known to peak in other species (Pérez-Rodríguez et al., 2005; Lau et al., 2015; Lu et al., 2022). We chose diploid *T. thalictroides*, tetraploid *T. dioicum*, and high-level polyploid *T. dasycarpum* (24×) as representative species. *Thalictrum thalictroides* is in clade I and has the ancestral traits of insect pollination and hermaphrodite flowers, with petaloid sepals that contain conical cells. In contrast, *T. dioicum* and *T. dasycarpum* from clade II are wind-pollinated polyploids with small and inconspicuous unisexual flowers that contain long pendulous stamens or plumose stigmas on long styles (Soza et al., 2012; Martínez-Gómez et al., 2023).

There was a trend towards high *Thalictrum* paleo*MIXTA* expression for specific paralogs in the carpels of the three species in the leaves of *T. dioicum* carpellate (female) plants and in the sepals of carpellate *T. dasycarpum* flowers (**Figure 1B**). Stamens and sepals from staminate (male) flowers showed a lower combined relative expression across the three species. *T. thalictroides* had expression in all organs analyzed, likely from a combination of having conical–papillate cells in some organs and trichomes in others, with the highest expression in carpels, followed by sepals, leaves, and stamens. Similarly, both paralogs from *T. dioicum* showed a high carpel-specific expression, suggesting partial redundancy in function. In *T. dasycarpum*, the expression was low to undetectable in the leaves, and one paralog (*TdaPMX1b.2*) had a high expression in carpels while another (*TdaPMX1a*) had a higher expression in stamens. On closer inspection, the R2 and R3 MYB domains of the lowest-expressing copy (*TdaPMX1b.1*) had two single amino acid substitutions, K43N and S76N, in the R2 and R3 domain, respectively (**Supplementary Figure S1**). Single amino acid substitutions in the R2R3 domains can cause loss of promoter–site interactions in other MYB proteins, resulting in the downregulation of expression (Hichri et al., 2011; Dai et al., 2016). Taken together, specific paleo*MIXTA* paralogs showed a sex-dependent expression pattern in dioecious *Thalictrum*, with the lower-level polyploid *T. dioicum* showing an overlapping expression of paralogs and the higher level polyploid *T. dasycarpum* exhibiting more specialization of paralog expression patterns.

### Targeted gene silencing reveals a leaf trichome morphogenesis role for paleoMIXTA

In order to investigate the gene function in a paleo*MIXTA* representative from an early-diverging eudicot, *Tth*paleo*MIXTA* (*TthPMX*) was targeted for downregulation by VIGS in *T. thalictroides*, on its own or together with *PHYTOENE DESATURASE* (*PDS*, as a “reporter” causing visible tissue photobleaching). The infection efficiency was 35%, with 15 out of 45 plants showing a range of photobleaching between 3 to 4 weeks after infiltration. Plants treated with the empty vector (EV = mock control) were indistinguishable from untreated wild-type plants, except for the previously described background effect of small necrotic lesions (Di Stilio et al., 2010) (**Supplementary Figure S2**). Viral transcripts (TRV1 and TRV2) were detected in treated plants and EV controls (**Supplementary Figure S3A**).

**Figure 2:**
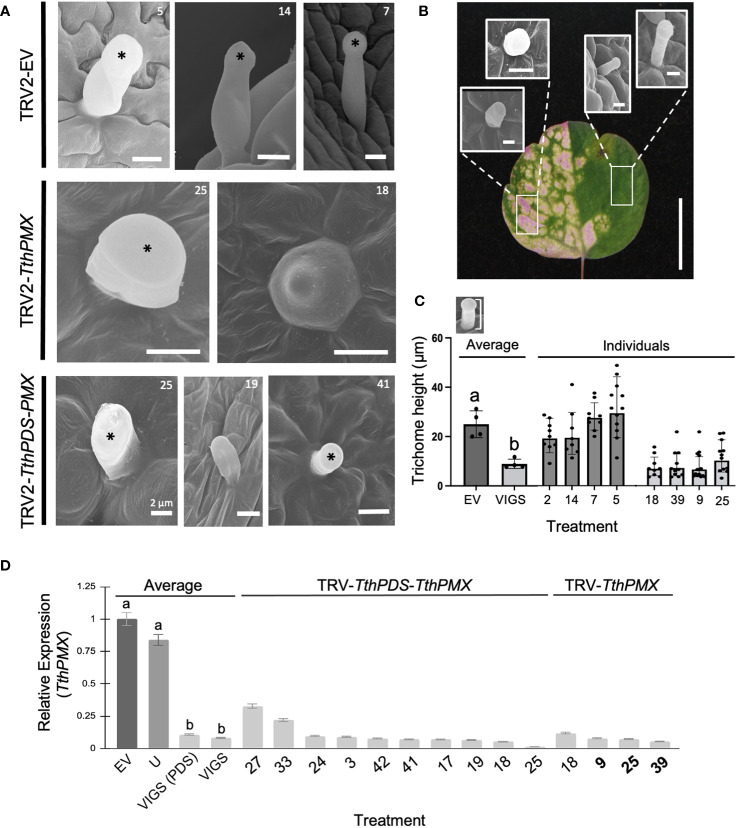
Targeted silencing of a *Thalictrum* paleo*MIXTA* ortholog affects leaf trichome morphogenesis. **(A)** Comparison of trichomes found on the abaxial epidermis of leaves of *Thalictrum thalictroides* in three representative empty vector control plants (TRV2-EV, top row) and four VIGS-treated plants with the construct TRV2-*Tth*paleo*MIXTA* (*TthPMX*). Asterisks indicate the position of the trichome head. Reduced “stubby” trichomes in plants undergoing targeted gene silencing for *TthPMX* represent three independent transgenic lines (middle and bottom row, transgenic line numbers shown for cross-reference). Scale bar = 10 μm, except as indicated. **(B)** Representative trichome phenotypes in green and photobleached sectors within a variegated leaf undergoing partial silencing of *TthPDS*. Rectangles show areas where the trichomes were sampled. Scale bar = 10 mm. **(C)** Quantitative difference in trichome cell height, measured as diagrammed in the top inset. Different letters indicate statistical significance between group means in one-way ANOVA with Tukey’s HSD (*P* ≤ 0.0001, *N* = 4 independent transgenic plants). **(D)** Molecular validation of VIGS experiments by qPCR for *TthPMX* as average expression level relative to *TthACTIN* and *TthEEF1* housekeeping genes, normalized against the empty vector controls. Error bars represent ± standard error of the mean. Different letters indicate statistically significant differences in one-way ANOVA with Tukey’s comparison test (*P* = 0.008).

Targeted gene silencing was validated by qPCR, showing an approximately 11-fold decrease in the average expression of *TthPMX* in plants treated by VIGS compared to empty vector controls (**Figure 2D**). Fully photobleached and variegated (partially photobleached) leaves from plants treated with TRV2–*ThPDS*–*ThPMX* showed a correlated downregulation of both genes, confirming the utility of *ThPDS* as a visual indicator of gene silencing (**Supplementary Figure S3B**).

To further establish the role of *T. thalictroides* paleo*MIXTA* in leaves, we quantified the epidermal features on their adaxial and abaxial epidermis from VIGS-treated plants, including the density and height of trichomes, the stomatal density, and the pavement cell shape. *T. thalictroides* has sparse trichomes on the abaxial leaf epidermis (**Figures 2A, B**). Under scanning electron microscopy (SEM), the trichomes appeared unicellular and capitate (enlarged toward the tip), which places them in the glandular category based on morphology (Simpson, 2019; Werker, 2000). The trichomes did not differ between the leaves of empty vector control and untreated plants (**Figure 2A**, top row). In contrast, the trichomes on the leaves of VIGS-treated plants showed either a “stubby”, short-stalk phenotype (**Figure 2A**, middle and bottom rows) resembling a conical cell or one that appeared irregular in shape (**Figures 2A, B**; **Supplementary Figure S4**).

**Figure 3:**
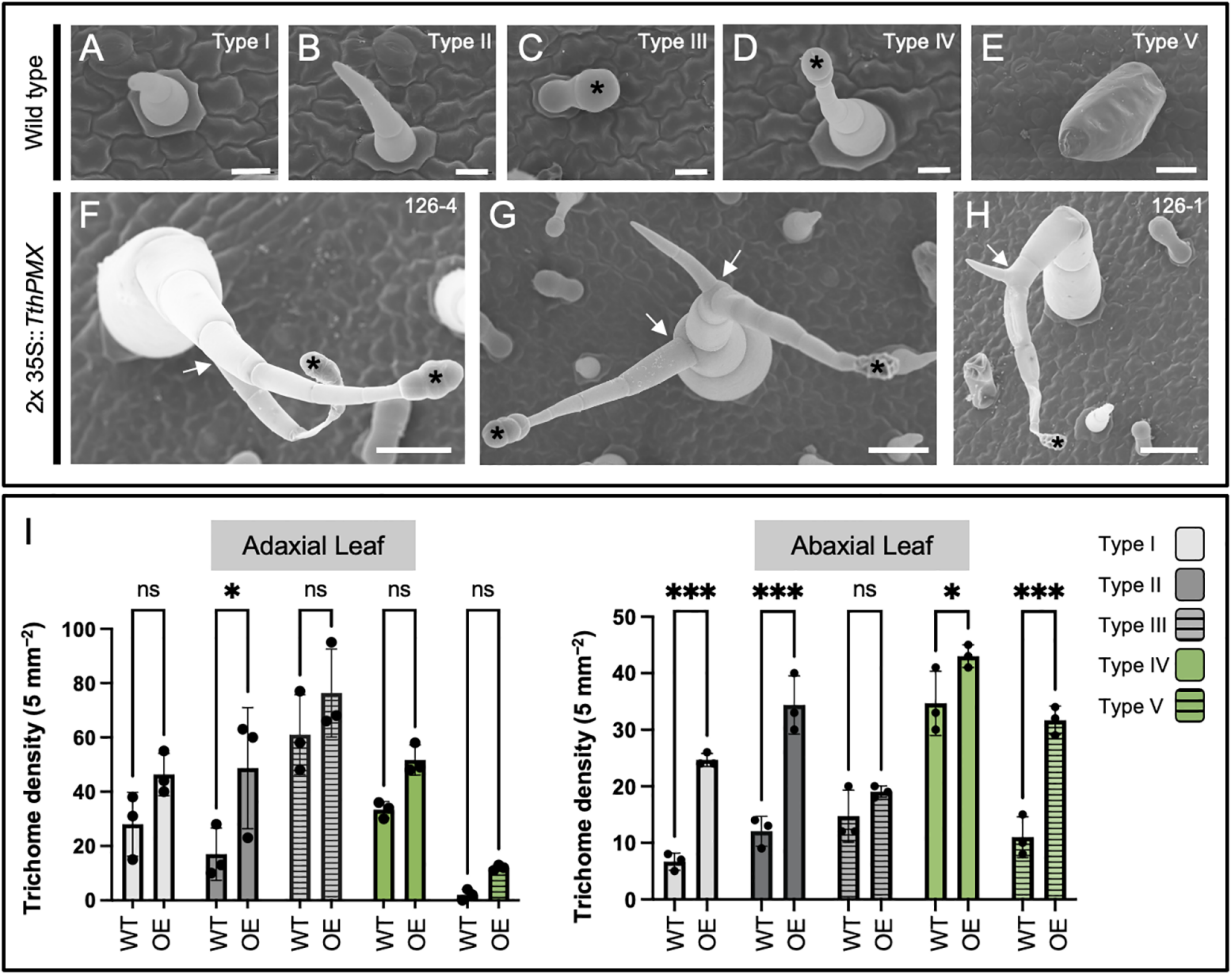
Overexpression of the *MIXTA* family ortholog *TthpaleoMIXTA* from *Thalictrum thalictroides* (Ranunculaceae) in tobacco leaves leads to ectopic and abnormal trichomes. **(A–E)** Trichome types I–V on the leaves of wild-type tobacco. Black asterisks indicate the position of the glandular head of capitate trichomes. **(F–H)** Ectopic branched trichomes on transgenic tobacco leaves; white arrows indicate abnormal branching. **(I)** Average trichome density ± SE by trichome type per 5 mm² on adaxial (left) and abaxial (right) leaf epidermis from three independent transgenic tobacco lines (2x35S::*TthPMX*) compared to wild type. Asterisks indicate statistically significant differences in trichome counts in a two-way ANOVA with Holm–Šídák’s multiple comparisons. ns, not significant; *P < 0.05; ***P < 0.001. Scale bar = 50 μm.

To further confirm that trichome developmental abnormalities resulted from the downregulation of the target gene, we compared sectors of photobleached and green tissues within variegated leaves (**Figure 2B**). Green leaf sectors had qualitatively taller trichomes than those found in photobleached sectors (**Figure 2B**; **Supplementary Figure S4**). To further quantify this observation and to avoid the potential side effects of PDS downregulation, we measured the trichome height in single-construct VIGS (without *PDS*); the average trichome was 2.7-fold shorter in VIGS-treated leaves compared to the empty vector controls (**Figure 3C**, *p* ≤ 0.0001, *N* = 41). Taken together, these results suggest that *T. thalictroides* paleo*MIXTA* plays a role in the elongation and morphogenesis of leaf trichomes.

We investigated additional epidermal features that have been reported to be influenced by other *MIXTA* family members, such as pavement cell morphology and stomatal density (Brockington et al., 2013). No significant qualitative differences were observed in the morphology of pavement cells between VIGS and control plants (**Supplementary Figure S5A**). Because stomatal density appeared to vary along the leaf, we sampled three regions on the abaxial side of transgenic plant leaves: top, middle, and base. In spite of some variations in stomatal density (visualized as a heat map, **Supplementary Figure S5H**), there was no statistically significant difference neither among leaf regions for a given treatment (**Supplementary Figures S5I, J**) nor between treatments when comparing averages (**Supplementary Figure S5G**, *N* = 5 independent transgenic plants per treatment).

### Heterologous overexpression of *Thalictrum* paleo*MIXTA* induces ectopic and branched trichomes in tobacco leaves

Heterologous overexpression was previously pursued using an established bioassay in tobacco to observe changes in ovary wall cell morphology (Di Stilio et al., 2009), and fixed leaves from that experiment were used here for observations of their epidermal features under SEM. In the tobacco family Solanaceae, glandular, non-glandular, and defense trichomes are found (Bar and Shtein, 2019). Two types of non-glandular (types I and II), glandular (III and IV), and one type of defense trichome (type V) were identified on the upper and lower epidermis of wild-type tobacco leaves (**Figures 3A–E**).

Transgenic plants overexpressing *TthPMX* exhibited ectopic types II and IV branched trichomes (**Figures 3F–H**, arrows) and a higher density of type II trichomes adaxially and of types I, II, IV, and V abaxially (**Figure 3I**). Taken together, these findings suggest that *TthPMX* is a positive regulator of trichome development, capable of affecting their morphogenesis and patterning on the leaf epidermis of a core eudicot.

### Identification of candidate genes in the leaf gene regulatory network of paleo*MIXTA*

**Figure 4:**
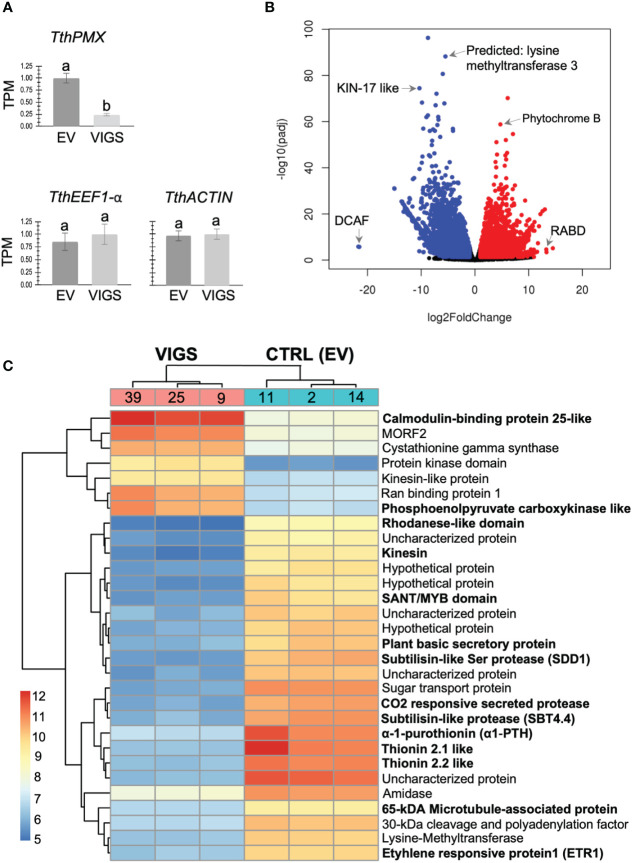
Identification of co-regulated candidate genes in the *Thalictrum* paleo*MIXTA* gene regulatory network. RNA-Seq analysis of differentially expressed genes (DEGs) in leaves from plants targeted for *TthPMX* downregulation (VIGS) vs. empty vector control (EV). **(A)** RNA-Seq validation with normalized count expression in transcripts per million of *Tth*paleo*MIXTA* and the housekeeping genes *TthEEF1-alpha* and *TthACTIN*. Means ± SE from three biological replicates; different letters indicate highly statistically significant difference (*p* < 0.0001) in one-way ANOVA followed by Tukey’s multiple comparisons. **(B)** Volcano plot depicting log fold change in expression; red indicates upregulated differentially expressed genes (DEGs) and blue downregulated DEGs. Identified candidate genes amongst outliers are shown. **(C)** Hierarchical clustering and heat map of the top 30 DEGs with the highest or the lowest expression profiles. The color scale indicates the expression levels (log_2_ fold change) relative to TRV2-EV controls. Genes identified via Diamond BLAST are listed; those of special interest are shown in bold and further discussed in the text.

To determine the molecular mechanisms underlying the role of paleo*MIXTA* in trichome development, we compared the transcriptomes of leaves from three mock-treated (empty vector) and three validated TRV2–*TthPMX* VIGS transgenic lines by RNA-Seq (**Figure 4**, transgenic line numbers in bold). There were 368,326,658 total reads, with a yield of 110,499 Mb and an average quality score of 35.84 (93.32% bases ≥3) (**Supplementary Table S3**). To independently validate the RNA-Seq results, we analyzed the expression counts for our target gene (*TthPMX*) and for genes known to have ubiquitous and stable expression (housekeeping genes). Leaves from VIGS-treated plants exhibited, on average, a fourfold decrease in *TthPMX* expression compared to controls, while the transcripts of the housekeeping genes *TthEEF1-alpha* and *TthACTIN* showed comparable expression levels in both treatments (**Figure 4A**).

The RNA-Seq analysis resulted in approximately equal numbers of significantly differentially expressed genes (DEGs) that were either upregulated (49%) or downregulated (50.3%) during the targeted silencing of *TthPMX*, using a cutoff log_2_ fold change of ±4, respectively. Differentially expressed genes were mapped onto a volcano plot, where the annotation of outliers identified a Ras-related protein (RABD) and phytochrome B that were significantly upregulated. Downregulated DEGs were identified as coding for DNA Damage Binding1, CULLIN4-associated factor homolog (DCAF), stress and UV damage response protein KIN-17-like, and lysine methyltransferase (**Figure 4B**). Annotation of the 30 highest- and lowest-expressing DEGs in the heat map uncovered a few more candidate genes upregulated in leaves undergoing VIGS of *ThPMX* (**Figure 4C**). These comprised orthologs of microtubule regulating *CALMODULIN-BINDING 25-LIKE* (*CaML-25*), kinesin-like protein (KLP), gluconeogenesis-associated phosphoenolpyruvate carboxy-kinase like (*PEPCK*), and multiple organellar RNA editing factor 2 (*MORF2*). The downregulated genes included those coding for motor proteins such as KINESIN and 65-kDa microtubule-associated protein (MAP65), defense peptides such as thionins (THI 2.1, THI 2.2, and α-1-purothionin), Rhodanese-like domain, epidermal specific subtilisin-like protease (SDD1; Von Groll et al., 2002), SANT/MYB domain proteins involved in chromatin remodeling, and ETHYLENE RESPONSIVE FACTOR 1 (ETF1) expressed highly in *Arabidopsis* mature trichomes (Jakoby et al., 2008). Taken together, the comparative transcriptomic analysis suggests that *TthpaleoMIXTA* influences trichome morphogenesis via the regulation of genes coding for candidate proteins involved in microtubule regulation, epidermal cell patterning, plant defense, and chromatin remodeling.

### *Thalictrum* paleo*MIXTA* affects conical–papillate cell development in floral organs

#### VIGS leads to loss of conical–papillate cells on showy stamen filaments of *Thalictrum clavatum*

**Figure 5:**
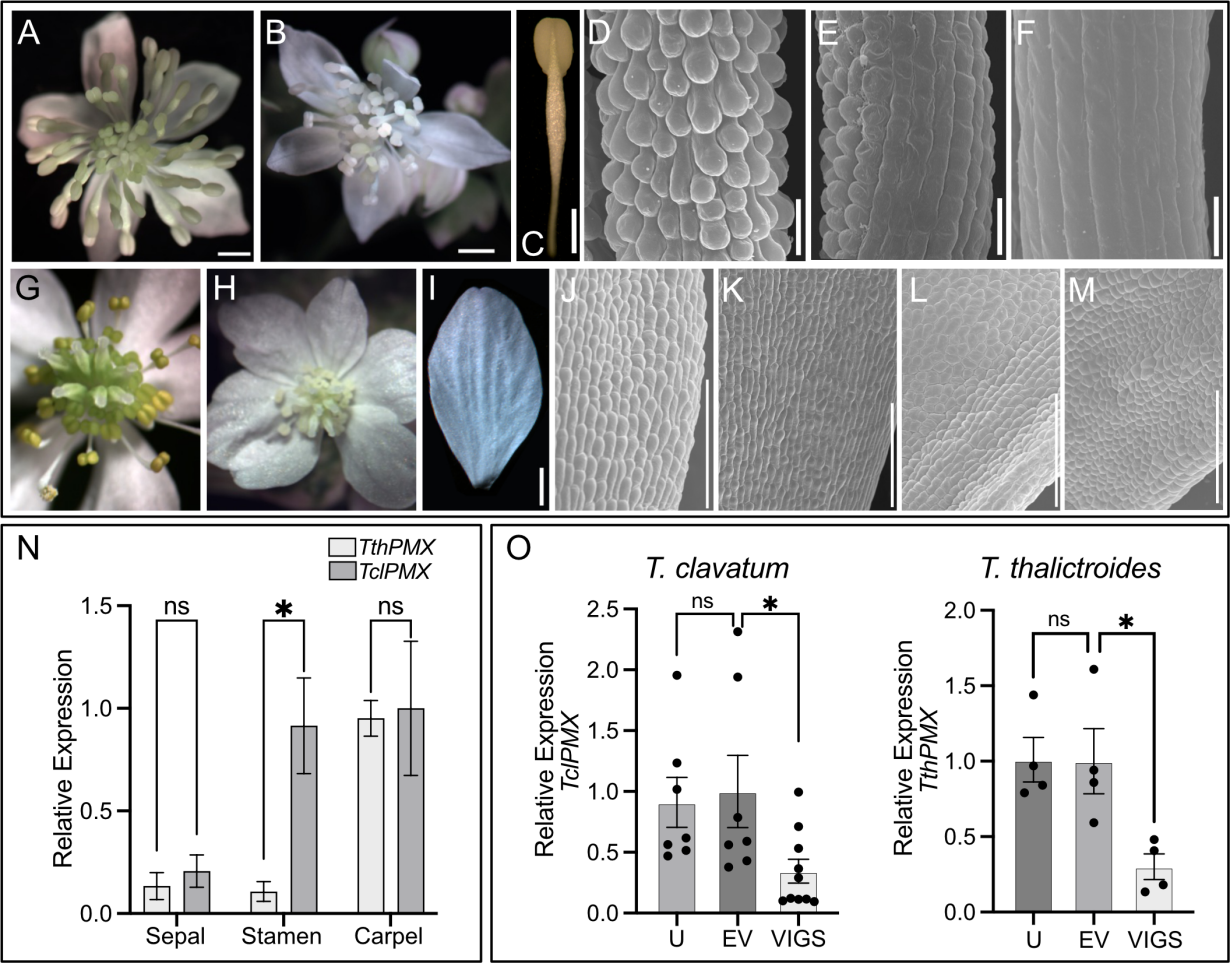
*Thalictrum* paleo*MIXTA* orthologs promote conical–papillate cells on the epidermis of flower organs. Virus-induced gene silencing (VIGS) of *ThPMX* in *Thalictrum clavatum* **(A–F)** and *T. thalictroides* **(G–M)**. **(A)** *T. clavatum* whole untreated flower. **(B)** VIGS-treated flower showing signs of photobleaching in the central region. **(C)** Control (untreated) outer stamen, whole view. **(D)** Detail of stamen filament in **(C)**, with protruding conical–papillate cells. **(E)** Stamen filament detail of flower treated with TRV2–PDS–*TclPMX*, showing signs of incomplete gene silencing; note the conical–papillate cells on the left half compared to the flatter cells on the right half. **(F)** Fully silenced stamen filament with flat epidermal cells. All filaments depicted are from fully mature outer stamens (before anther dehiscence). **(G)** *T. thalictroides* whole untreated flower. **(H)** VIGS-treated flower showing signs of photobleaching in the central region (immature stamens and carpels). **(I)** Control (untreated) outer sepal, whole view. **(J, K)** Detail of the adaxial epidermis of two untreated sepals with slightly protruding conical–papillate cells. **(L, M)** Detail of sepal adaxial epidermis undergoing VIGS in flowers from two independent plants treated with TRV2–*TthPDS*–*TthPMX*, with flatter epidermal cells. **(N)** Wild-type relative expression by qPCR of *TthPMX* and *TclPMX* in dissected floral organs of *T. thalictroides* and *T. clavatum*, respectively. **(O)** Molecular validation of VIGS experiments in *T. clavatum* and *T. thalictroides*. U, untreated; EV, empty vector control; VIGS, virus-induced gene silencing. The values shown in **(N, O)** are average expression levels relative to *TthACTIN* and *TthEEF1* housekeeping genes, normalized to the highest expressing organ in **(N)** (carpel) or to EV control in **(O)**. The asterisk denotes statistical significance at *P* < 0.05 by Dunnett’s multiple-comparisons test, ns: not significant. The scale bar is 1 mm in **(A–C, G–I)** and 100 µm in **(D–F, J–M)**.

To further investigate the potential role of *ThPMX* in the epidermal features of flowers, we conducted functional studies by VIGS in two species of *Thalictrum* representing distinct flower morphologies, the “petaloid sepal” and “showy stamen” morphotypes (Martínez-Gómez et al., 2023). Untreated *T. clavatum* flowers are white to light pink and consist of a few small sepals and numerous stamens with showy or “petaloid” filaments (clavate, flattened, and wider at the top, and colored) as previously described (Di Stilio et al., 2010) (**Figure 5A**). Photobleaching of PDS is detectable in the floral center as a sign of effective gene silencing (**Figure 5B**). Conical–papillate cells are most prominent in the filaments of outer stamens (**Figures 5C, D**) and also present adaxially in sepals. In order to test the role of *TclPMX* in the conical–papillate cells of *T. clavatum* stamens, we used VIGS and observed the resulting floral cellular phenotypes under SEM. In wild-type plants, this gene is expressed at low levels in sepals and mostly in stamens and carpels, whereas *T. thalictroides* expresses relatively less in stamens (**Figure 5N**).

As in the leaf experiments, plants were visually assessed for photobleaching in leaves and flowers post-infiltration. *T. clavatum* began to show signs of photobleaching in leaves 13 days after infiltration, and the first buds opened 4 days later. The photobleached flowers tested positive for the TRV1 and TRV2 viruses as expected in all 10 plants that displayed the phenotype. The mock-treated plants displayed an identical TRV1 as well as a lower-molecular-weight TRV2 band, confirming the absence of insert (**Supplementary Figure S6**). The untreated plants did not show signs of TRV1 or TRV2 (**Supplementary Figure S6A**). Target gene silencing was validated by qPCR, showing a significant downregulation of approximately threefold in VIGS treatments compared to controls (*P* = 0.04). The SEM of stamens showed epidermal cells that looked flatter in VIGS treatments, either partially (**Figure 5E**) or fully along the filament (**Figure 5F**), and these observations were confirmed in the stamens of at least three independent transgenic plants.

#### VIGS leads to loss of conical–papillate cells on the petaloid sepals of *Thalictrum thalictroides*

Untreated *T. thalictroides* flowers have white petaloid sepals, stamens that are green to yellow, and carpels that are green toward the base (**Figures 5G, I**), therefore allowing for the detection of photobleaching in the flower center (**Figure 5H**). The conical–papillate cells are subtle in this species but can be observed adaxially at the base of the sepals under SEM (**Figures 5J, K**). Flowers showing signs of photobleaching similarly contained TRV virus (**Supplementary Figure S6B**), and the qPCR of dissected sepals showed a significant downregulation of *TthPMX* by approximately threefold (*P* = 0.02, *N* = 4 independent transgenic plants) (**Figure 5O**). Their sepal epidermis had flatter cells adaxially than the untreated controls (compare **Figures 5J–M**).

Thus, these results confirm a role for *TthPMX* in conical–papillate cells on the perianth in the non-core eudicot *Thalictrum*, previously suggested via heterologous expression in tobacco (Di Stilio et al., 2009). Taken together, the *T. clavatum* experiments suggest a more general function for *ThPMX* in epidermal protrusions beyond the perianth and into the reproductive organs.

## Discussion

### Evolutionary history of *Thalictrum MIXTA* family

Within the early-diverging eudicots, *Thalictrum* is in the order Ranunculales, sister to the rest of the eudicots. Since *MIXTA* and *MIXTA-like* are found exclusively within the core eudicots, we propose here that *Thalictrum* orthologs represent a “paleo” *MIXTA* lineage, analogous to how the *APETALA3* subfamily of floral MADS-box genes has a paleo*AP3* lineage in early-diverging eudicots sister to the euAP3/TM6 core eudicot gene duplication (Kramer et al., 1998). Therefore, *Thalictrum* paleo*MIXTA* is informative of the ancestral function of *MIXTA* family orthologs.

The phylogenetic inference of the *MIXTA-like* family in *Thalictrum* uncovered a lineage-specific duplication that coincides with a WGD event in the genus. This result suggests that, early in the history of the genus, the evolution of the *MIXTA-like* gene family was impacted by polyploidy, which is ubiquitous in *Thalictrum*.

### *T. thalictroides PMX* is a positive regulator of leaf trichomes

The MYB family R2R3 SBG9-A gene *TthPMX* (previously *TthMYBML2*) from *T. thalictroides* had been shown to increase the height of ovary epidermal cells when overexpressed in tobacco (Di Stilio et al., 2009). This study provides evidence for the role of *TthPMX* in conical–papillate cells in the floral organs of *Thalictrum* while uncovering a novel function as a positive regulator of leaf trichome development. Most subgroup 9-A *MIXTA* family genes such as *Antirrhinum majus* (snapdragon) *AmMYBML1* (Pérez-Rodríguez et al., 2005) and *A. annua AaMIXTA1* (Shi et al., 2018) are positive regulators of trichome development. In tomato, the ectopic expression of *SIMIXTA* increases type I trichomes, whereas RNA silencing reduces them (Ewas et al., 2016; Schuurink and Tissier, 2020). However, certain *MIXTA-like* genes have developed inhibitory roles in trichome initiation, such as *Mimulus guttatus MgMYBML8* (Scoville et al., 2011) and *Arabidopsis MYB106* (Gilding and Marks, 2009). Hence, our finding that *Thalictrum* paleo*MIXTA* positively affects trichome development suggests that the negative role of MIXTA-like genes in trichome development could be a derived function in certain core eudicots.

### *Thalictrum* paleo*MIXTA* modulates trichome morphogenesis via microtubule remodeling

*Arabidopsis* has become a model to study the morphogenesis and anisotropic development of epidermal cells (Yang et al., 2019). Microtubule binding proteins are important components of the plant cytoskeleton that have been implicated in the development of both conical cells and branched trichomes (Reddy et al., 2004). Previously, trichomes with short stalks were observed in *Arabidopsis* loss-of-function mutants for kinesin-like calmodulin-binding protein (KCBP) (Oppenheimer et al., 1997). However, the mechanisms of trichome morphogenesis in species outside of this derived model plant are poorly understood.

Our RNA-Seq data shows the downregulation of genes coding for the microtubule motor protein KINESIN, DCAF, KIN-17-like, and MAP65 and the upregulation of microtubule-regulating CaML-25 and KLP. In *Arabidopsis*, calmodulin-like proteins interact with kinesin microtubule motor proteins to regulate trichome morphogenesis (Oppenheimer et al., 1997; Reddy et al., 2004). In addition, there was downregulation of subtilisin-like proteases such as SDD1 that play a role in cell signaling and spacing of stomatal cells (Von Groll et al., 2002); however, their role in the spacing of other epidermal cells is unknown. While our qualitative SEM analysis of VIGS-treated plants did not uncover statistically significant differences in the morphology of pavement cells or the stomatal density, there was visible variability in stomatal densities among individual transgenic plants across the top, middle, and bottom leaf abaxial regions (**Supplementary Figure S5H**), thus warranting further investigation.

The top candidates included DEGS that are also highly expressed in mature *Arabidopsis* trichomes (top 5%), such as *PEPCK*, *ETF1*, *KINASE*, and *RABD*. These results together suggest that *MIXTA* family orthologs from phylogenetically distant plant species may have converged in regulating cell elongation in trichomes through similar microtubule-mediated mechanisms. Comparable genetic components point to the potential conservation of the genetic machinery for the development of epidermal extensions in eudicots.

### A potential role for *Thalictrum* trichomes in plant defense

*Thalictrum* species are often cultivated for their wealth of biochemical compounds such as alkaloids, found in roots and leaves (Hao et al., 2021). It is currently unknown whether *Thalictrum* trichomes have the ability to synthesize or accumulate bio-compounds. Our study provides evidence that targeted silencing of *TthPMX* affects trichome morphology, and modulates genes related to plant stress and defense. Basic secretory protein (BSP), Rhodanese-like domain, multiple enzymes and genes encoding antimicrobial thionins were co-repressed in transgenic plants undergoing gene silencing. Glandular trichomes of *Artemisia annua* secrete Rhodanese-like domain protein for defense and detoxification (Wu et al., 2012), while leaf specific-thionins are small metabolites with roles in plant defense against pathogens and herbivores (Escudero-Martinez et al., 2017). Therefore, it appears likely that *Thalictrum* trichomes play a role in defense against herbivores. Moreover, the downregulation of secondary metabolites such as secretory proteins, sugar transport proteins and enzymes like methyltransferase, amidase, and protease in VIGS-treated plants supports a glandular role for *Thalictrum* trichomes that is consistent with their morphology (**Figure 2**).

### A gene with many functions: *MIXTA family* genes regulate the development of multiple epidermal forms in different plant systems

It has been speculated that timing, developmental stage, or localization may determine the specialized cell forms that develop from the expression of *MIXTA-like* genes (Pérez-Rodríguez et al., 2005). Interestingly, our comparative transcriptomic approach revealed that *TthPMX* positively regulates a SANT/MYB domain-containing protein involved in local chromatin remodeling (Boyer et al., 2004), suggesting that paleo*MIXTA* genes could potentially reprogram epidermal cell fate via the regulation of chromatin remodeling complexes.

Functional studies of *MIXTA* family orthologs in the monocot and core eudicot clades suggest that they are sufficient to drive the development of multiple epidermal features. For instance, in the orchid *P. aphrodite*, both *PaMYB9A1/2* paralogs can drive the differentiation of conical cells and cuticle biosynthesis in petals (Lu et al., 2022). Similarly, snapdragon *AmMYBML1* can modulate the differentiation of trichomes in leaves and of conical cells in petals (Pérez-Rodríguez et al., 2005). In the liverwort *Marchantia polymorpha* (a bryophyte, or non-vascular plant), *MIXTA* family homologs play a role in cuticle in addition to papillate cells, representing a more ancestral function potentially common to all land plants (Xu et al., 2020).

Here, we show that *Thalictrum paleoMIXTA* from the early-diverging eudicots can modulate the development of leaf trichomes and floral conical–papillate cells. Hence, if the trichome and conical cell roles found here were extended to other early-diverging eudicots, it may be possible to reconstruct a combined function back to the last common ancestor of eudicots. In closing, our findings in *Thalictrum* join those in other plant systems to suggest a deeply conserved role for *MIXTA* family transcription factors in trichomes and conical–papillate, readily producing a diversity of epidermal features.

## Methods

### Plant materials

*T. thalictroides* bare root plants were purchased from Sundquist nursery (Poulsbo, WA) and mature *T. clavatum* plants from Gardens of the Blue Ridge Inc (Pineola, NC). Both sets of plants were vernalized at 4°C before infiltration. Voucher specimens are listed in **Supplementary Table S1**.

### Phylogenetic analysis of *Thalictrum MIXTA* family genes

*MIXTA* family homologs were recovered from 23 species representative of all major clades in the *Thalictrum* phylogeny by PCR and cloning and/or direct sequencing (vouchers listed in **Supplementary Table S1**). Genomic DNA was extracted using the MP Bio101 FastDNA Kit, and complete or near-complete paleo*MIXTA* loci were amplified by PCR with locus-specific primers (**Supplementary Table S2**) and cloned with pCRII TOPO TA (Invitrogen, Carlsbad, CA, USA). Positive clones were purified using a 5 Prime FastPlasmid Miniprep kit and sequenced (Genewiz, South Plainfield, NJ, USA). At least eight colonies per diploid species or 15 colonies per polyploid species were sequenced to encompass all paralogs. The sequences were edited in Sequencher version 4.9 (Gene Codes Corporation, Ann Arbor, MI, USA) and aligned using MUSCLE (Edgar, 2004). INDELS were coded as gaps and missing data was coded as “?”. The models of evolution for each data set were determined using jModelTest version 2.1 (Posada, 2008). The GTR+I+Γ model was selected based on the Akaike Information Criterion (Akaike, 1974). Bayesian inference analysis was conducted using MrBayes v.3.2 (Ronquist et al., 2012) with a parallel MCMC analysis of 50 million generations, sampling every 1,000 generations. Convergence was checked using the average standard deviation of split frequencies (<0.01) and the effective sample size (ESS) values (>200). The first 25% of trees were discarded as burn-in. The remaining trees were pooled to construct a 50% majority rule consensus tree and visualized using FigTree v1.4.4 (Rambaut et al., 2018).

### Gene expression analysis

To determine paralog-specific expression, real-time quantitative PCR (qPCR) was carried out on a Bio-Rad CFX qPCR system, and the results were analyzed in BioRad CFX Manager3.0 software (Bio-Rad laboratories, Hercules, CA, USA). Locus-specific primers were designed in NCBI’s Primer3 (**Supplementary Table S2**), and primer specificity was tested via melting curve analysis, resulting in a single peak per primer set. Quantification of expression for *TthPMX*, *TthPDS*, *TclPMX*, *TdiPM1b*, *TdiPMX2a*, *TdaPMX1a*, *TdaPMX1b.1*, *TdaPMX1b.2*, and *TdaPMX2b* was performed as previously described (Galimba et al., 2018). Briefly, each 10-μL reaction contained 5 μL of SYBR Green PCR Master Mix (Bio-Rad, Hercules, CA, USA), 0.5 μL (10 μM) of locus-specific primers, 1 μL of template cDNA, and 3 μL of water. The samples were amplified for 40 cycles in duplicate, including a no-template control. The reactions were normalized to the *Thalictrum* orthologs of two housekeeping genes, *ACTIN* and *EEF1* (*EUKARYOTIC ELONGATION FACTOR 1*), using the 2^-ΔΔCT^ relative quantification method (Livak and Schmittgen, 2001). The standard deviation of Ct values of reference genes was calculated, and the average of three technical replicates was used to ensure minimal variation in gene expression. Average values and standard errors were graphed and compared statistically by single-factor ANOVA followed by Tukey’s comparisons or Dunnett’s multiple-comparisons test.

### Construct preparation

*T. clavatum* and *T. thalictroides* share 99.8% nucleotide identity for PDS, allowing us to use the same PDS sequence across the two species. *TthPDS* had been previously cloned into TRV2 (Di Stilio et al., 2010) and was used here as a marker in a double construct with the target gene, also previously cloned (Di Stilio et al., 2009). Briefly, the TRV2–*TthPDS* construct was double-digested with XbaI and BamHI and then ligated to a *TclPMX* or *TthPMX* 400-bp fragment, half of which comprised the end of the C terminal and the other half the 3′UTR. The target sequences were obtained by PCR amplification from plasmid DNA containing the whole coding region of *TthPMX* (previously *TthMYBML2*, Di Stilio et al., 2009) using primers with added restriction sites (**Supplementary Table S2**).

### Virus-induced gene silencing

The infiltration of *T. thalictroides* and *T. clavatum* was carried out as previously described (Di Stilio et al., 2010) with minor modifications. Briefly, TRV1, TRV2–*TthPMX/TclPMX*, and TRV2–*ThPDS*–*ThPMX* starter cultures were grown overnight with selective antibodies and subsequently used to inoculate 500-mL cultures. *Agrobacterium* was centrifuged at 4°C and 4,000*g* for 15 min before being resuspended in an infiltration medium (10 mM MES, 20 μM acetosyringone, and 10 mM MgCl2). Each culture was resuspended to a final OD600 of 2.0 and incubated for 3 h at room temperature. Islet L-77 (Lehle Seeds, Round Rock, TX, USA) was added at 100 uL/L as a surfactant.

Dormant tubers of both species that had been kept at 4°C for 3 weeks were removed from soil and rinsed, and a small razor blade incision was made away from the meristem before being submerged in an infiltration medium containing a 1:1 ratio of TRV1 and TRV2 cultures. *T. thalictroides* tubers were treated with a construct with the gene of interest alone or with both the target gene and the marker gene phytoene desaturase (*ThPDS*), whose downregulation induces photobleaching. *T. clavatum* tubers were treated with the double construct only. In total, 40 tubers were vacuum-infiltrated per species and then potted in 2.5″ Deepots™ (Stuewe & Sons, Tangent, OR, USA) using Sunshine Mix 4 soil (Sun Gro, Bellevue, WA, USA). The plants were kept in growth chambers under a 16-h light–dark cycle at 22°C/18°C. An additional 15–20 plants per species were infiltrated with empty TRV2 (mock). This control was used to test for background viral effects. A total of 15 untreated plants were grown under the same conditions as an additional control.

### Molecular validation of VIGS experiments

Fully expanded mature leaves and open flowers before anther dehiscence were collected from plants at 3 to 4 weeks post-infiltration, flash-frozen with liquid nitrogen, and stored at -80°C. Total RNA was extracted using the Spectrum Plant Total RNA Kit or Trizell reagent (Invitrogen, CA, USA) according to the manufacturer’s protocol. The samples were treated with DNAase I (Thermo Scientific™, Waltham, MA, USA), and first-strand cDNA was synthesized using iScript cDNA synthesis kit (Sigma, Burlington, MA, USA). cDNA was synthesized using TRV1- and TRV2 -specific primers (for viral detection by RT-PCR) or the manufacturer’s mix of random and polyT primers (for qPCR) and amplified using a 51 × 30 PCR cycle and locus-specific primers (**Supplementary Table S2**). The RT-PCR products were run on 1.2% agarose gel and photographed on GelDoc Image lab software package (Bio-Rad, Hercules, CA, USA).

To determine if target genes had been successfully downregulated, qPCR was conducted as explained above with *ThPDS*- and *ThPMX*-specific primers (**Supplementary Table S2**) on leaves or flowers (whole or dissected) at 4 to 10 samples each of untreated, mock (empty vector), and treatment. Average values and standard deviations for each biological replicate were graphed relative to the highest-expressing tissue.

### Phenotypic analysis

Plants of both species were photographed using a Nikon D3400 hand-held camera and a dissecting microscope (Nikon SMZ800, Nikon Instruments Inc., Melville, NY, USA) equipped with a QImaging MicroPublisher 3.3 RTV digital camera (Surrey, BC, Canada).

#### Preparation of samples for scanning electron microscopy

Fully expanded mature leaves and open flowers were collected whole or dissected and fixed in 10% formaldehyde, 5% acetic acid, and 50% alcohol (FAA) for 1 h at RT and overnight at 4°C, dehydrated through an alcohol series, critical point-dried, mounted on stubs, and sputter-coated with gold. Observations were made on a JEOL NeoScope JCM-7000 (University of Washington Microscopy Facility). The leaves fixed in FAA from a previously published tobacco bioassay overexpressing *TthMYBML2* (here renamed to *TthPMX*) under a strong constitutive promoter (Di Stilio et al., 2009) were similarly prepared for SEM. The images were assembled in Affinity Publisher (Serif (Europe) Ltd) or Microsoft Powerpoint.

#### Quantification of epidermal features

*T. thalictroides* leaves and dissected floral organs were photographed under SEM. For leaf trichomes, a 1-mm^2^ surface adjacent to the mid vein was sampled adaxially and abaxially in three regions: top, middle, and base. Trichomes were found only on the abaxial side and were measured from the base to the tip with Image J (Schneider et al., 2012) using either a straight or segmented line from four independent transgenic plants (at eight to 14 trichomes each).

Tobacco leaf trichomes were sampled in a 5-mm^2^ area at the base adjacent to the mid vein both adaxially and abaxially. The trichome type was identified, and the number of trichomes found for each type (I–V) was averaged per treatment (three independent transgenic plant lines per treatment). Statistical significance was determined using a two-factor ANOVA followed by Holm–Šídák’s test for multiple comparisons. Chimeric trichomes such as those with two or more types represented within one individual trichome were counted as a separate category and removed from the count data to avoid redundancy.

### Comparative transcriptomic analysis

Four biological samples of *T. thalictroides* leaves were collected and validated from mock-treated and VIGS-treated lines (TRV2–*TthPMX*), and their total RNA was extracted for the RNA-Seq analysis. Sequencing was outsourced to Azenta-Genewiz (Burlington, MA, USA), where sample quality was evaluated in an Agilent 2100 Bioanalyzer (Agilent Technologies, Inc., CA, USA) before library preparation. mRNA sequencing was conducted using polyA selection on a HiSeq 4000, and the adapters were removed from the sequence reads with Trimmomatic v.0.36 (Bolger et al., 2014). The resulting reads were mapped to a *de novo Thalictrum thalictroides* transcriptome, assembled with Trinity v2.5 (Haas et al., 2013). Open reading frames were identified with EMBOSS GetOrf, and the transcriptome assembly was annotated using Diamond BLASTx.

### Differential gene expression analysis (RNA-Seq)

To determine changes in transcript expression between controls and plants targeted for gene silencing, gene hit counts were used for downstream differential expression analysis (DEGs). Briefly, the standard bioinformatic analysis package (Azenta-Genewiz, South Plainfield, NJ, USA) consisted of DeSeq2 to generate normalized hit counts, Wald test to generate *p*-values for statistical significance (Wald, 1943) and log_2_ fold changes to quantify the expression change between groups. The Benjamini–Hochberg test (Benjamini and Hochberg, 1995) was used to generate adjusted *p*-values. Genes with adjusted *p*-values <0.05 and absolute log_2_ fold change >1 or ≤1 were regarded as differentially expressed. Significant DEGs were functionally annotated, and transcripts of interest, such as *TthPMX* and orthologs of the reference genes *EEF1-alpha* and *ACTIN*, were identified in the assemblies using BLAST. Transcript normalized counts (transcripts per million) were used to graph their relative expression as a validation step. The average expression for three bio-replicates was normalized to mock-treated controls (empty vector, EV), and the statistical significance was calculated using single-factor ANOVA and Tukey’s test for multiple comparison.

## Data availability statement

The datasets presented in this study can be found in online repositories. The names of the repository/repositories and accession number(s) can be found below: SRA data: PRJNA1030785, NCBI SRA PRJNA1030785 and DRYAD dataset https://doi.org/10.5061/dryad.tdz08kq5k.

## Author contributions

SZ: Writing – review & editing, Formal Analysis, Investigation, Visualization, Writing – original draft. AS: Investigation, Writing – review & editing. VD: Writing – review & editing, Conceptualization, Funding acquisition, Supervision.
