# Supplementary material for: A eudicot MIXTA family ancestor likely functioned in both conical cells and trichomes
Source: Front Plant Sci. 2023 Dec 18;14:1288961. doi: 10.3389/fpls.2023.1288961 (PMC10764028; doi:10.3389/fpls.2023.1288961)
Supplement: Supplementary file 1 [file DataSheet_1.pdf]

Supplementary Materials

for

A eudicot *MIXTA* family ancestor likely functioned in both conical cells and trichomes

Simra Zahid, Anjelique F. Schulfer and Verónica S. Di Stilio

Content List:

Suppl. Figure 1: Amino acid alignment of *Thalictrum* paleo*MIXTA*.
Suppl. Figure 2: Phenotypes of leaves undergoing VIGS.
Suppl. Figure 3: Molecular validation of leaf VIGS.
Suppl. Figure 4: Trichome phenotype in VIGS variegated leaves.
Suppl. Figure 5: Stomatal density of VIGS treated leaves.
Suppl. Figure 6: Molecular validation of flower VIGS.
Suppl. Table 1: Voucher information.
Suppl. Table 2: Primers.
Suppl. Table 3: RNA-seq sample sequencing statistics.

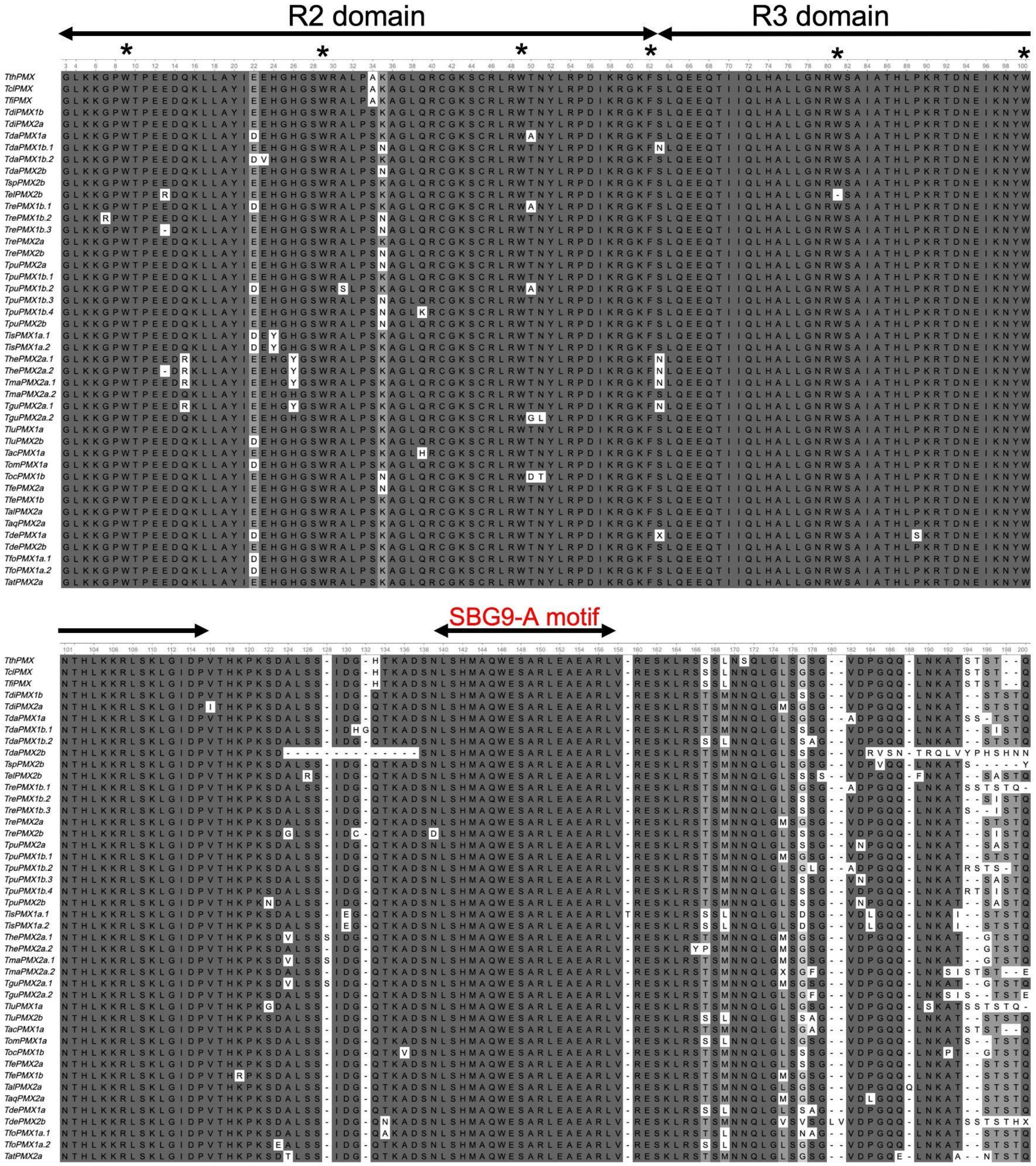

Suppl. Figure 1: Amino acid alignment of R2R3 SBG9-A MYB *Thalictrum MIXTA* orthologs from *Thalictrum* (Ranunculaceae). Conserved R2, R3 and Subgroup 9A domains are indicated. Dark gray shading indicates identity, light gray similarity, dashes gaps, and asterisks conserved (regularly spaced) tryptophan residues.

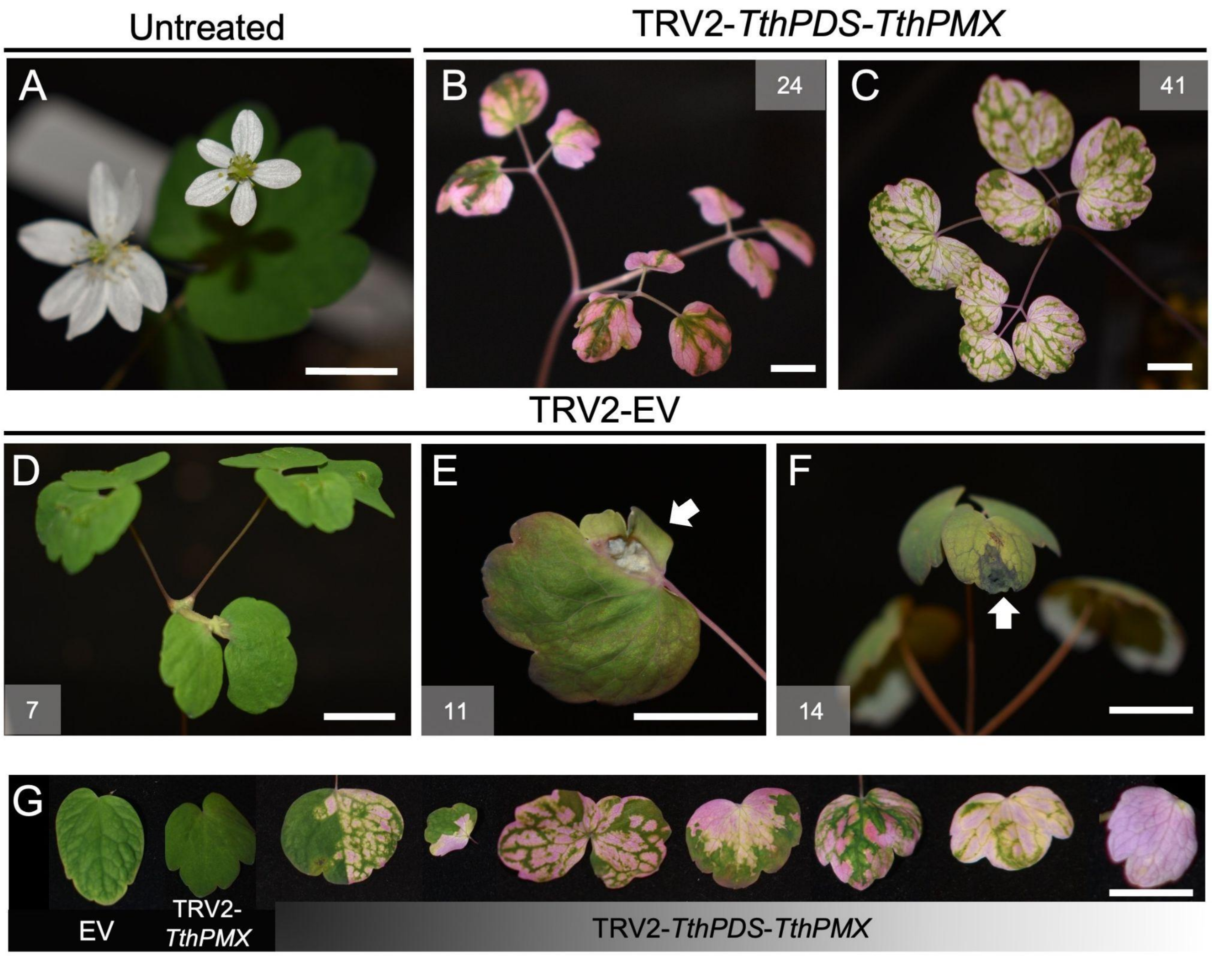

Suppl. Figure 2: Phenotype of leaves undergoing Virus Induced Gene Silencing (VIGS) of a *MIXTA* family ortholog from the early diverging eudicot *Thalictrum thalictroide*s (Ranunculaceae). *PHYTOENE DESATURASE* (PDS) results in photobleaching of photosynthetic tissue, acting as a "reporter". (A) Untreated control (B-C) TRV2-*TthPDS*-*TthPMX* treated plants showing photobleaching of leaf tissue. (D-F) Mock-treated plants (empty TRV2 vector, or EV), arrows show background viral effect of tissue necrosis. (G) Leaf profiles of treated groups, from left to right: EV control, TRV2-*TthPMX*, and TRV2-*TthPDS*-*TthPMX* showing a range of photobleaching resulting in variegated leaves. Scale bars: 10 mm.

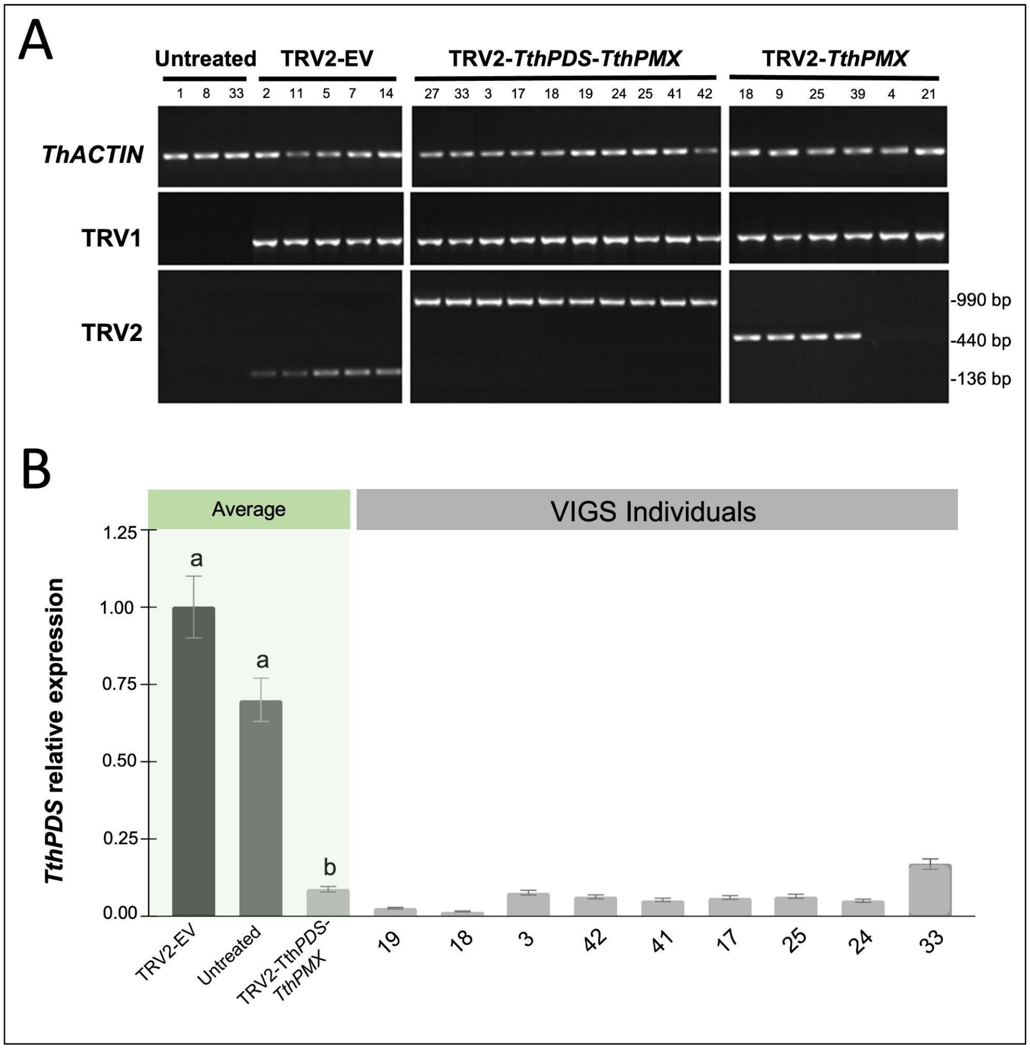

Suppl. Figure 3: Molecular validation of targeted silencing of *TthPMX* by VIGS in *T. thalictroides*. (A) Reverse Transcriptase (RT) PCR with locus specific primers detects viral transcripts in leaves of TRV2-EV, TRV2-*PMX*, and TRV2-*PDS-PMX* transgenic plants (numbers correspond to independent transgenic line numbers). *TthACTIN* used as a loading control. Viral transcripts were only detected in treated lines. Approximate band size indicated for TRV2; larger bands result from the presence of the targeted gene insert**.** (B) Gene expression validation by qPCR for *TthPDS,* normalized to empty vector controls. Expression is relative to *TthACTIN* and *TthEEF1*. *One way ANOVA, different letters indicate significant differences by Tukey's comparison test (p= 0.008). Error bars represent ± standard error of the mean.

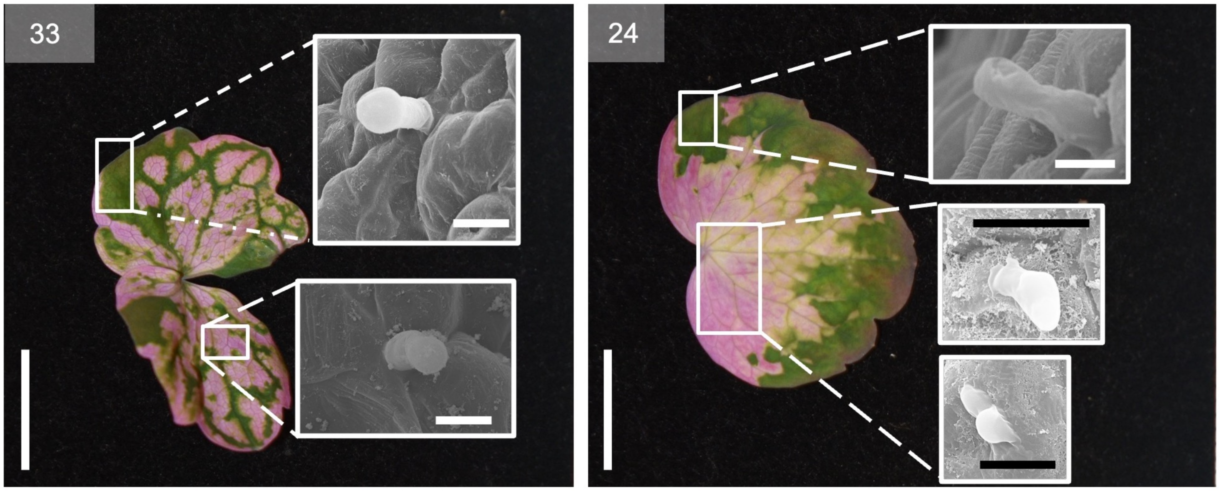

Suppl. Figure 4: Trichome phenotype in green and photobleached sectors of variegated leaves resulting from targeted silencing of *Thalictrum* paleo*MIXTA*.

Insets show scanning electron microscopy of representative leaf trichomes in two independent TRV2-TthPDS-*TthPMX* transgenic plants. Scale Bar= 10 mm (main panel), or 10µm (inset).

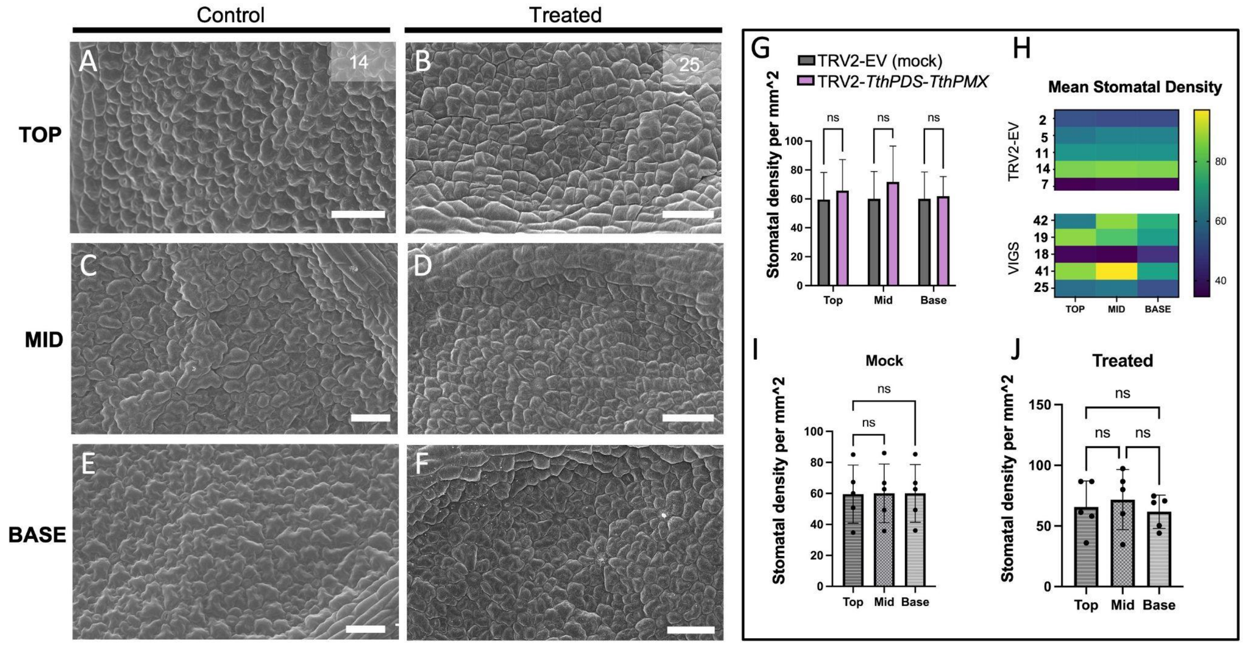

Suppl. Figure. 5: *Thalictrum* paleo*MIXTA-like* does not affect leaf stomatal density.

SEM of abaxial top, middle and base regions of leaves treated by VIGS of *TthPMX* and *PDS* (*PHYTOENE DESATURASE*, as reporter causing photobleaching of green tissues) and controls: (A, C, E) empty vector controls; (B, D, F) Leaves undergoing silencing of *TthPMX*. (G) Mean stomatal density per mm^2 across the leaf landscape in controls and VIGS-treated plants. (H) Heat map showing mean stomatal density at the top, middle, and base leaf sectors for five independent transgenic plants and empty vector controls (TRV2-EV). (I, J) Stomatal densities per mm^2 in control (Mock) and VIGS-treated plants (Treated). Means ± SE shown (n = 5). Scale bars in (A-F) = 50 μm.

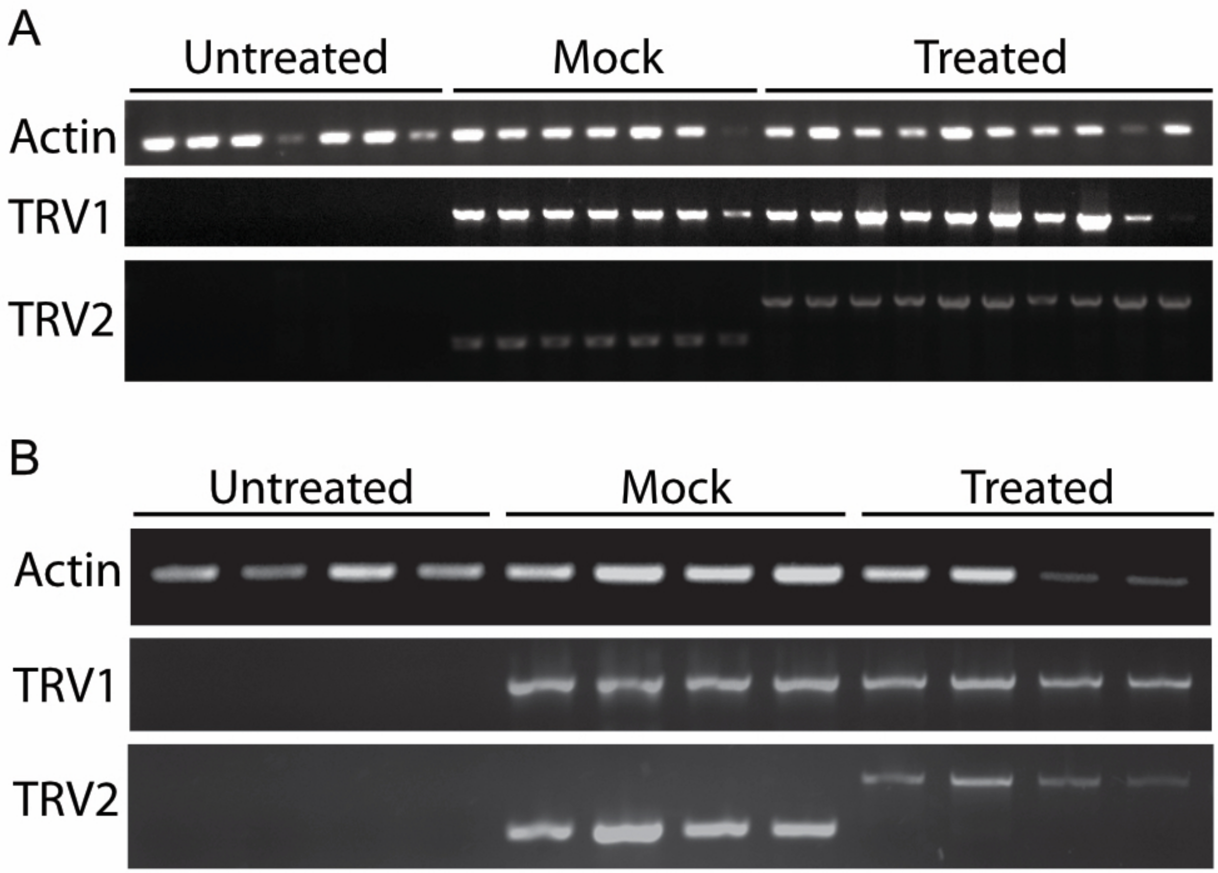

Suppl. Figure 6: Molecular validation of VIGS in floral tissue of *T. clavatum* (A) and *T. thalictroides* (B). Mock=empty vector. Treated =VIGS of paleo*MIXTA* ortholog.

Suppl. Table 1: Voucher information for taxa sampled for *MIXTA* family orthologs. A: Herbarium of the Arnold Arboretum, Harvard University; IDS=Idaho State University; ID=University of Idaho Stillinger Herbarium; OSC=Oregon State University; UWBG: University of Washington Biology Greenhouse; WIS=University of Wisconsin Herbarium; WTU=University of Washington Herbarium; YNUH: Herbarium of Yeungnam University**.**

**Species**	**Collector and collection number**	**Locality**	**Herbarium-accession**
Thalictrum actaeifolium Siebold & Zucc	S. Park 821-823	Mt. Mucheok, Gimhae-si, Gyeongnam, South Korea	YNUH
Thalictrum alpinum L.	V. Di Stilio 115	Cultivated from Ion Exchange Nursery, Iowa, USA.	WTU-367701
Thalictrum aquilegiifolium L.	V. Di Stilio 108	Cultivated at UWBG from Cricklewood Nursery, Washington, USA.	WTU-369715
Thalictrum atriplex Finet & Gagnep.	*Ho 2594*		TI

Thalictrum clavatum DC.	V. Di Stilio 127	Cultivated at UWBG from gardens of the Blue Ridge, North Carolina, USA.	WTU-379880
Thalictrum dasycarpum Fisch., C.A.Mey. & Avé-Lall.	V. Di Stilio 137	Cultivated at UWBG Medicinal Herb Garden from Prairie Moon Nursery, Minnesota, USA	WTU
Thalictrum delavayi Franch.	V. Di Stilio 121	Cultivated at UWBG from: B & T World Seeds, Aigues-Vives, France; Sundquist Nursery, Washington, USA.	WTU- 376543
Thalictrum dioicum L.	V. Di Stilio 101<br>M. Sain 60	Lithia Springs, South Hadley, Massachusetts, USA. U. of Wisconsin, Muir Woods, Madison, Wisconsin, USA.	A<br>WIS-v0398757 (carpellate)<br>WIS-v0398758 (staminate)
Thalictrum elegans Wall. ex Royle	V. Di Stilio 136	Cultivated at UWBG greenhouse from KBG seed (Nepal origin)	WTU
Thalictrum fendleri Engelm. ex A.Gray	V. Soza 192<br>V. Soza 1921	Cultivated at UWBG from Heronswood Nursery, Washington, USA; and USDA Ames29956 seed	WTU

Thalictrum filamentosum Maxim.	V. Di Stilio 104	Cultivated at UWBG from nursery (Heronswood, WA, USA).	WTU
Thalictrum foetidum L.	V. Soza 1923	Nursery, Arrowhead Alpines, Michigan, USA	WTU
Thalictrum guatemalense C.DC. & Rose	A. Liston 1124	Cultivated at UWBG from wild collected seed, Mexico, Mexico	OSC
Thalictrum hernandezii Tausch ex J.Presl	A. Liston 1125	Cultivated at UWBG from wild collected seed, Mexico, Mexico	OSC
Thalictrum isopyroides C.A.Mey.	V. Di Stilio 111	Cultivated at UWBG from Heronswood Nursery, Washington, USA.<br>Plant World seed	WTU
Thalictrum lucidum Gunther ex Lecoy.	V. Di Stilio 122	Cultivated at UWBG from Botanische Gärten der Universität Bonn, Germany.	WTU-376545

Thalictrum macrostylum Shuttlew. ex Small & A.Heller			unvouchered
Thalictrum occidentale A. Gray	Karen 63		TEX
Thalictrum omeiense W. T. Wang & S. H. Wang	A. Liston 1166	Cultivated at UWBG from Heronswood nursery, Washington, USA	OSC
Thalictrum pubescens Pursh	D. Baum & D. Howarth 375	Arnold Arboretum, Jamaica Plains, Massachusetts, USA.	A
Thalictrum revolutum DC.	R. Dale Thomas & S. Leslie 96982<br>G. D. Sones	Bradley County, Arkansas, USA.<br>Grand Rapids, Michigan, USA	IDS0006486<br><br>ID043114
Thalictrum thalictroides (L.) A.J.Eames & B.Boivin	V. Di Stilio 124	Cultivated at UWBG from Sundquist Nursery, Washington, USA	WTU-376542
Thalictrum sparsiflorum Turcz. Ex Fisch. & C.A.Mey	S. Ickert-Bond s.n.	Cultivated at UWBG from wild-collected seed. Fairbanks, Alaska, USA.	WTU-425454

Suppl. Table 2. Primers used for VIGS validation and cloning. EEF-1=Eukaryotic Elongation factor 1; PDS= Phytoene Desaturase; PMX= paleo*MIXTA*.

**Primer Name**	**Sequence**	**Description, use**
pTRV1_fwd =OYL195	5'-CTTGAAGAAGAAGACTTTCGAAGTCTC-3'	TRV1, VIGS validation RT PCR
pTRV1_rev =OYL198	5'-GTAAAATCATTGATAACAACACAGACAAAC-3’	TRV1, VIGS validation RT PCR
ThML2_VIGS_F	5'-GCTCTAGAAATACAGGCCTTCAAGATATGG-3'	Cloning of ThPMX VIGS fragment
ThML2_VIGS_R	5'-CGGGATCCTTGCCAGACAATTTGAGAATC-3'	Cloning of ThPMX VIGS fragment
qPCR_EEF-1alphaF4	5'-CTT CTT GCC TTC ACA CTT GGA GTC-3’	Reference gene, RT qPCR
qPCR_EEF-1alphaR4	5'-TGT TGT CAC CCT CAA ACC CAG AG-3’	Reference gene, RT qPCR
Tth Actin for2	5'-GCA GAA CGG GAA ATT GTC CGC-3'	Reference gene, RT qPCR
Tth Actin rev2	5'-CCTGCAGCTTCCATTCCGATCA-3'	Reference gene, RT qPCR
PYL156F	5’-GGTCAAGGTACGTAGTAGAG-3’	TRV2, VIGS validation RT PCR
PYL156R	5’-CGAGAATGTCAATCTCGTAGG-3’	TRV2, VIGS validation RT PCR

TthPDS_F_RT	5' - TGA ACA ACG ATG GAA CCG TG - 3'	VIGS Reporter, RT qPCR
TthPDS_R_RT	5' - GTC AGC ATA CAC ACT CAA AAG G - 3'	VIGS Reporter, RT qPCR
TthML2_qPCR_F3	5'-CAG TGA AAG AAG AAT GTG ATG AAG AGTA -3	*T. thalictroides PMX*, RT qPCR
TthML2_qPCR_R3	5'-TGA CTG GAA TGT GCT CGT TTC-3’	*T. thalictroides PMX*, RT qPCR
Tcl ML2 R	5' TGA CTG GAA TGT GTC CAT TAC 3'	*T. clavatum PMX*, RT qPCR
TdioT1F1_qpcr	5’ TGA AGA GTA TAT GGG TGG TGG T -3’	*T. dioicum* locus1, RT qPCR
TdioT1R1_qpcr	5’ TTG GAT GAT TCT GAC GCC CA -3’	*T. dioicum* locus1, RT qPCR
TdioT2F1_qpcr	5’ GTT CCT CCT ATC ATT AAT AGA G -3’	*T. dioicum* locus2, RT qPCR
TdioT2R1_qpcr	5' CAG AAT CAT CAT CAA AAG TTG GA -3’	*T. dioicum* locus2, RT qPCR
TdasyT1F1_qpcr	5’ GCT AAG CTC AAT TGA TCA CG -3’	*T. dasycarpum* locus1, RT qPCR
TdasyT1R1_qpcr	5’ CGT TGT GGC TGT GTG GAT A -3	*T. dasycarpum* locus1, RT qPCR
TdasyT2F2_qpcr	5’ AAG TTG CGG TCC AGT TCA TT -3’	*T.dasycarpum* locus2,

		RT qPCR
TdasyT2R2_qpcr	5’ TCA GAT CAA GCG AAG TCA TAG -3’	*T.dasycarpum* locus2 specific, qPCR
TdasyT3F1_qpcr	5’ CTG GTC TGC TAA CAG GTG AAG GG -3’	*T.dasycarpum* locus3 specific, qPCR
TdasyT3R1_qpcr	5’ AAC ACC TTC AGA AGT GCC TGA TGG -3’	*T.dasycarpum* locus3 specific, qPCR
TdasyT4F1_qpcr	5’ CAG TAC TGG TGG TGG CCT TGA T- 3’	*T.dasycarpum* locus4 specific, qPCR
TdasyT4R1_qpcr	5’ GCA AAG GCA TAG GGT TAT CGC -3’	*T.dasycarpum* locus3 specific, qPCR
ML2ForDeg2	5’ TCT CTT CTG CTT CAC CAC TTC -3’	Degenerate, *MIXTA* family cloning
ML2RevDeg2	5’ATT AAT KTG ATT GAG CTT GTC AGG T -3’	Degenerate, *MIXTA* family cloning
TthMYBML2_IntF	5'-TTC AGA GGT GTG GAA AGA GTT-3'	*Thalictrum* MIXTA family, gDNA cloning internal primers
TthMYBML2_IntR2	5'-GCT TTG ACC AGR TTC CTT GC-3'	*Thalictrum* MIXTA family, gDNA cloning internal primers

Suppl. Table 3. Sequencing statistics for RNAseq experiments.

Sample ID	Barcode Sequence	# Reads	Yield (Mbases)	Mean Quality Score	% Bases >= 30
EV2	TAAGGCGA+CTTAATAG	46,589,130	13,977	35.86	93.42
EV14	TAAGGCGA+TCGCATAA	46,526,090	13,958	35.87	93.49
EV11	TAAGGCGA+ATAGCCTT	40,386,460	12,116	35.83	93.32
VIGS9	CGTACTAG+TCTTACGC	46,156,446	13,847	35.80	93.10
VIGS25	CGTACTAG+ACTCTAGG	46,089,467	13,827	35.84	93.27
VIGS39	CGTACTAG+AGCTAGAA	48,789,517	14,637	35.77	93.05
